# A Paper upon Diseases of the Teeth

**Published:** 1858-04

**Authors:** W. W. Allport


### ARTICLE III.

*A Paper upon Diseases of the Teeth.* Read before the Cook Co. Medical Society at Chicago, and published at their request. By W. W. ALLPORT, D. D. S.

MR. PRESIDENT AND GENTLEMEN,—In obedience to your request, I appear before you, not to deliver a lecture, but to say a few plain words about some of the diseases of the teeth and their effects upon the general health. While I feel my own inability to do justice to the subject, I thank you, on behalf of my profession, for this recognition of ourselves as co-workers with you in the noble field of medicine.

The tendency of men to quackery, has always made your profession look with a jealous eye upon new divisions of medical labor. We do not blame you for your tardiness in recognizing the value of dental surgery; for, as the guardians of the people, you were bound to be watchful, where experience had taught that the people love quackery in medicine as they love fanaticism in religion. We have patiently waited, and having passed through the ordeal of your scrutiny, I thank you, on behalf of my profession, for this expression of your generous confidence.

I take it for granted, you do not expect me to describe the details of operative dentistry, in which you could have but little interest; I shall, therefore, confine my remarks to the nature and causes of *some* of the diseases of the teeth, and their effect upon the health of patients—a subject which requires the watchful care of the physician not less than the skill of the dentist.

When a physician listens to a lecture on medical jurisprudence, he does not expect to become a lawyer; he simply desires to become familiar with some of those points of law which describe his duties and the mutual rights of himself and patients. On this principle, I take it for granted you do not desire to learn dentistry, but rather to gain that information which will enable you to avoid doing injury to the teeth of your patient, and also to judge when it is necessary to call to your aid the services of a competent dentist.

I will pass over the subject of first dentition with the remark, that where you are called to cut the gums, in order to facilitate this physiological process, the duty might, with propriety, be performed by us, for most of the diseases of these organs are the result of bad habits, formed in early life; so we cannot too early impress the parent's mind the necessity of care for their children's teeth, and this can be done by no one so well as the dentist.

As the *decay* of the teeth is the *disease* which attracts the most attention, and is also most fatal in its results, the question arises, What is the disease called "decay of the teeth?" The theory of some of the most able practitioners of the past—such as Fox, Tomes, Bell and Jourdain—was, that the decay of the tooth was caused by something which suspended or destroyed the vital action. Some supposed that the application of heat and cold injured the enamel, which laid the foundation for serious disease. Others thought that decay was produced by a lateral pressure of the teeth against each other; and others thought the use of calomel destroyed the constitution of the teeth, thus producing disease. This last theory has become one of those popular fallacies which quackery has used without stint against your profession. In all these it was a common idea that decay sometimes began under the enamel, upon the surface of the bone. For lack of time I shall pass over all these theories, except those of inflammation and calomel, by saying, that, like many other wise notions of the past, they are now laid away in the tomb of fogyism.

The theory of inflammation was, doubtless, the result of a supposed similarity in the structure of the teeth and the other bones. This reasoning would at first seem plausible. Upon this point Prof. Harris says:

"This inference, it must be confessed, to one who has not made the disease of the former a subject of close and critical investigation, would seem to be irresistible. But observation has proven it, so far as most of the diseases of the teeth are concerned, to be incorrect. By instituting a comparison between caries of the teeth and that of other bones, it will at once be perceived that there is not the slightest analogy between the disease as it occurs in the one and manifests itself in the other. In the former, it consists simply of a decomposition of the earthy basis of the organs; whereas, in the latter, it is analogous to ulceration in soft parts, and constantly discharges a fetid sanies, and throws out granulations of fungous flesh. These are phenomena which dental caries never exhibit, and they establish a wide difference between it and the disease as occurring in the other osseous structures of the body."

"If inflammation of the bony structure of the teeth were the cause of caries, the disease would be as likely to develop itself in one part of the tooth as in another. The root, the interior of the crown, between the pulp cavity and the enamel, would as frequently be the part first attacked as the the exterior surface."

Instead of the decay of teeth commencing *under* the enamel, as it very likely would, were it at all analogous to the disease of the other bones, it is a fact, well known to every dentist, that it *never commences* under the enamel, but always makes its appearance upon the outside, and insidiously works its way to the dentine, and thence to the pulp-cavity.

"So far as is known, the blood vessels are the essential seat of inflammation."* Inflammation consists of a change of condition of these vessels, accompanied with heat, redness and swelling of the parts. I think it is regarded as a correct theory, that true inflammation cannot exist in a tissue which does not admit red corpuscles of blood. Thus the cornea of the eye, though subject to a variety of diseases, cannot be regarded as in a true state of inflammation until vessels carrying corpuscles of blood *extend into it.* In ordinary bone the average size of the tubuli is 1-500, and its smallest size 1-2000 part of an inch in diameter. The average size of the red corpuscles of blood is 1-4500 part of an inch, which, not being one half the size of the smallest tubuli of bone, this portion of the blood can readily ramify every part of the bone and its inflammation and suppuration can be accounted for upon the theory of common inflammation. But the physical structure of the dentine forbids the idea that inflammation is *ever* the cause of its decay ; for we learn that the largest tubuli in the dentine is only 1-10,000 part of an inch in

* Williams' Principles of Medicine, page 200.

diameter; and it is plain that it is impossible for these corpuscles of double the size to pass into the dental tubuli. Unless there is some other principle upon which to account for inflammation, it is impossible for *true* inflammation to exist in the bones of the teeth.

Calomel, which has long been the scape-goat for the sins of your profession, as a *direct* agent, has no effect whatever upon the bones of the teeth. I shall speak of it hereafter as a medicinal agent, which may lead to conditions of the system that may cause decay of the teeth.

From this it is plain that we mnst look to some other cause than inflammation for this disease.

The analysis of the tooth shows that the enamel consists of 99-100 parts of mineral and 1-100 part of animal matter, 94 parts of which are lime. The dentine contains 72-100 of mineral to 28-100 of animal matter. Sixty-nine parts of which are lime. With so large a proportion of the tooth made up of mineral matter, we should naturally look for its destruction, either in mechanical or chemical causes. From the nature of the case, it is very rare that mechanical causes can produce the effect. Experiments of a satisfactory character have proved that *acids* are almost universally the actual cause of decay.

The destruction of the teeth, as the result of external corrosive agents was not generally believed until within forty years, and not fully demonstrated until about 1842, when Prof. Westcott, by a series of experiments, tested it fully. This opinion, so completely demonstrated by him, is now adopted by the dental profession.

To demonstrate the effect upon the teeth of some of the acids in common use, as condiments and medicines, I have filled six open mouthed vials with acids, as I am assured by the druggist from whom they were procured, of the average strength used for medicinal and table purposes, and placed them in a water bath, kept at blood heat, for twenty-four hours. In each I have placed five teeth.

One tooth has remained 15 minutes.
" " " " 30 "
" " " " 1 hour.
" " " " 9 hours.
" " " " 24 "

We will now note the result.*

I venture the assertion, that in the city of Chicago, containing over 100,000 inhabitants, there are not 500 over five years of age, who do not take into their mouths, or manufacture in their systems, one or more of these acids, that is brought into direct contact with the teeth every twenty-four hours.

* The following are the results of acids upon the teeth, as exhibited to the Cook County Medical Society, by Dr. Allport, dentist. These notes were necessarily taken in great haste, and may be slightly incorrect, but they are accurate enough for all practical purposes. THOMAS BEVAN, *Secretary.*

| TIME. | SULPHURIC. | HYDROCHLORIC. | NITRIC. | AQUA REGIA. | CITRIC. | AROMATIC SULPH. | ACETIC. |
|---|---|---|---|---|---|---|---|
| 15 minutes | Enamel bleached. | Enamel slightly bleached. | Enamel easily removed with the nail. | Enamel slightly affected. | Enamel slightly roughened. | Enamel bleached. | Enamel but slightly affected. |
| 30 minutes | Roughened. | Roughened. | Nearly destroyed | Considerably softened. | Softened. | Slightly roughened. | Perceptible effect. |
| 1 hour | Softened. | Easily cut | Destroyed. | Easily removed. | Easily cut | Considerably softened. | Slightly bleached. |
| 9 hours | Surface easily removed. | Much softened. | • | Enamel entirely destroyed | Readily removed. | Easily removed. | Roughened. |
| 24 hours | Nearly destroyed | Easily removed. | Tooth entirely destroyed. | | | Mostly destroyed | Removed with a knife. |

I presume that there is not one of you who is not in the habit of giving to your patients hydrochloric, sulphuric, nitric, citric, or acetic acid, nearly every day. It is true that you use the precaution of giving it through a tube, and if *you* were the patient, due care would be taken that it did not come in contact with your teeth. But you are aware of the carelessness of most patients in the use of medicine, so that this precaution is of little avail unless taken under your own eye. Permit me to cite a case, which is only one of many, that have occurred under my own observation.

In August, 1856, a young lady, of seventeen years of age, called upon me to examine and attend to her teeth. I found them of full ordinary density of structure. I filled nine cavities with gold, and dismissed her, with her teeth in excellent condition, requesting her to keep them clean, and call upon me once in four months. At the expiration of this time she called, I found all the fillings in perfect order, and one new cavity to fill. I filled it, and told her I did not think she would require my services for a long time to come, but, as a precaution, desired her to see me as often as once in four months. In March following she went East, and being delayed longer than she expected, was thoughtful enough to write me, and ask if she had not better call upon some dentist to examine her teeth. I recommended her to an excellent operator, and after a thorough examination he pronounced her teeth in excellent condition. I mention these facts to show you that the constitution of her teeth was good, and not of that perishable kind which decay rapidly. The last of May, this young lady was taken sick, and unable to return home until September. She then called upon me, and to my surprise, I found her teeth in a deplorable condition ;—so much decayed that it was beyond the power of my art to restore them to health. I advised her to use them for a time as they were, and then have them replaced by artificial teeth. Upon asking her what medicine she had taken, she said she did not know, but that one kind was something *sour*, taken through a glass tube. You will agree with me that it was a very sad experience of acids taken through a tube.

Hydrochloric acid is found in imperfect gastric juice. Food ejected from the stomach, after it has been commingled with this acid, acts with great energy upon the teeth, the enamel becomes *roughened,* thus making a lodging place for foreign substances.

Acetic acid, as you have seen, acts readily upon the teeth, and from its general table use, and its manufacture in the mouth, by the fermentation of vegetable and animal substances, lodged about the teeth, is probably the most common destructive agent.

Saliva, in a healthy state, is alkaline, and no doubt is beneficial to the teeth by neutralizing whatever acid it may come in contact with, in the mouth. The secretions of the mucous membrane are naturally acid, and when this membrane is excited, and the secretions increased, if there is no healthy saliva to neutralize this, and it is left to combine with fermented food lodged around the teeth, the decay must be rapid.

It is not to be expected that the dentist can have much influence over these conditions ;—the most he can do, is to point out their injurious effect upon these organs, and I hold it to be the physician's duty, that where he has reason to believe the saliva is vitiated, he should make examination with test paper, and do all he can to correct the evil. I would suggest, that in addition to the precaution of taking acids through a tube, you should advise your patients, in such cases, to rinse their mouths with some alkaline solution, and thereby, in some degree, avoid the danger of this powerful chemical agent. As soon as the patient has been restored to health, the physician should see to it, so far as he is able, that his patient visits some competent dentist, so that no time may be lost in having whatever injury the teeth may have suffered, remedied as soon as possible.

I have said, that as a direct agent, calomel has no effect upon the teeth ;—that serious, and even fatal results to these organs are sometimes produced, I presume none of you will doubt:—but these effects are more often the result of the *injudicious* use of the drug, than a necessary result of its *judicious* use. The effect of calomel upon the system causes the gums and periosteum to inflame, and by absorption the necks of the teeth are left bare, and foreign substances are lodged between and around portions of the teeth not protected by enamel, and unless great care is taken to remove them the decay is rapid and inevitable. The absorption oftentimes is so great as to cause the teeth to loosen and drop out. Calomel, also, by vitiating the saliva, acts upon the teeth. The truth, however, is, that calomel is not guilty of one-fiftieth part of the injuries to teeth charged upon it. The popular feeling against it has been so stimulated by the denunciations of quackery, that if a patient has taken calomel at any time in his life, the decay of his teeth is traced to this cause. He may have used acids by the quart, and kept up a regular laboratory for their manufacture by food lodged around the teeth, and yet, *calomel—calomel* is the guilty one. It is but another illustration of the old adage, "Put a *lie* on horseback, and all the honest men in the world cannot chase it down;" and I am sorry to say that some of my profession have been foolish enough to pander to this popular prejudice.

Perhaps there will be no better place to say a few words about the proper means of cleansing the teeth. It is by no means uncommon for the patient to answer the dentist's appeal to use the brush, by the objection: "Dr. —— says it injures the teeth to *brush* them—a cloth is better—the rough brush causes the gums to bleed, and wears off the enamel." Such advisers forgot that a healthy gum will not bleed sooner than the tongue or lips, and that when they do bleed, it shows the necessity for depletion. The cause of this inflammation is the deposition of tartar, or other irritating substances, about the necks of the teeth, and under the free edges of the gums. There is the same necessity for its removal, as in the case of any other foreign substance, which causes irritation, and must be accomplished by mechanical means. When tartar has accumulated it should be removed by the dentist. The brush and the toothpick are the proper instruments to keep them clean, and it will be a triumph in the work of preservation when our patients learn that every tooth has four sides and a top, and that the vigorous and systematic use of these instruments are requisite for their cleanliness. If cleanliness were thus practiced, and the food carefully removed from the teeth after each meal, with the teeth perfectly formed, and the secretions healthy, decay would seldom occur.

From the fact that some teeth are of a hard firm texture, every seam or crevice in the enamel perfectly united, while others are of a comparatively soft or porous texture, leaving openings where agents may readily find their way to the dentine, there is a corresponding difference in their liability to decay. The difference arises from the lack of a proper amount of bony matter being secreted at the time of the formation of the teeth. From the fact that the vital organization of the teeth is low, if once imperfectly formed, they will remain with little change through life. The other parts of the body, being more highly organized, may in after life make up what its feeble energies failed to deposit in early life. We sometimes find teeth of very different constitution in the same mouth;—while one tooth was forming, there was an abundant supply of bony matter, and it cut its way through the gum in perfect condition; while another was in process of formation, the system may have been morbidly excited, the functions imperfectly performed, the supply of bony matter stopped, and assimilation checked; such a tooth can only be saved with the most scrupulous care on the part of the patient, and delicate and thorough manipulation of the dentist.

The administration of phosphate of lime to the mother, during the period of gestation and lactation, has been practiced by Drs. Taft and Watt, of Cincinnati, as they think, with great success. I take the liberty to suggest, that whenever there is reason to believe, either from the mother's health the condition of the teeth of either parent, or from children previously born, that good will result from such an experiment, it would be well to make the trial and carefully note the result. It is a new field, but from the experiments already tried, results have been obtained which will warrant further investigation.

I have not detailed to you all the causes of the decay of the teeth, for this would require a paper too long for your patience. If the theory, that external, corrosive agents are the proximate cause of this decay, is *correct*, then the *absolute cleanliness* of the parts, and the correction of any tendency to vitiated secretions in the mouth, are the only rational means for its prevention.

Having considered as fully as the limits of my time will permit, and yet as briefly as a due regard to the importance of the subject will justify, some of the causes which affect the teeth, I trust you will not think it improper, or outside of the *legitimate province* of dentistry, if I ask the attention of the Society to a brief, but general consideration of some of the influences and effects which the teeth and gums, when diseased, may have on other parts of the body and upon the general health.

An eminent writer upon this subject says:

"As the body is a unit, knit by the closest bonds, pervaded by one system of blood vessels and nerves, directed by one intelligence, and kept in a continual relation of reciprocal functions by an all-pervading law of reciprocal reaction and sympathy—as diseases of other parts, and these which, in distinction to well defined and limited affections, we call general, are capable of affecting the teeth, it might be apparent, if we had no particular facts in evidence, that the morbid condition of the teeth may produce corresponding evils in other parts, and may even involve the whole system in troubled and morbid action.

"It might also be evident that severe and long-continued pain, located in the immediate vicinity of the brain, and in parts little accessible to soothing appliances, cannot be less dangerous to health than pain in other organs, situated at greater distance from the nervous centres, and more easy of access.

"It might also be perceived that sensitive organs, in immediate contact with the great lining membrane of the thoracic and abdominal cavities, and intimately connected with it by function, cannot be less capable of propagating disorder to it, than parts located far from it, and having no immediate relation to it."

And yet as natural as these inferences are, and as important as they must appear to every intelligent and reflecting mind, you will pardon me, when I express the opinion that this subject does not receive that attention from the medical profession which its great importance demands. Until quite recently, the question, whether, physiologically or pathologically considered, these organs sustained any relation to the *hygienic* condition of other parts of the system, seems to have been almost entirely overlooked by physicians.

In cases of protracted ill health, the causes and conditions of which baffled the research and skill of the most intelligent in the profession, how few, in their earnest endeavors to improve the condition of their patients, ever pay the respect of a "hasty glance" at the *teeth* and *gums*, even when their morbid condition can hardly escape recognition by more senses than one. And when, at last, from anguish, their patients are driven to the dentist, as they suppose, for relief from local pain, and a mouthful of decayed teeth is removed, and ulcerated gums take on healthy action, how positive and rapid oftentimes the improvement in the general health. Cases illustrative of this, I doubt not, are within the experience and observation of nearly, if not quite, every member of this Society; and yet, very few would, I apprehend, in their daily practice, ever look to the teeth or gums of their patients, for the cause of their continued ill-health, until every source, real or imaginary, had been inquired into, in the, perhaps, vain attempt to find it.

If then, in such cases, the primary or predisposing cause is so readily, and I may say frequently overlooked, how much more likely is the agency of such influences to escape the attention or detection of the physician, in cases of recent occurrence, and when the disease assumes a type and form readily recognised and acknowledged. Surely, if *unsought* for in the one, it would be *unthought* of in the other.

I would not be understood as asserting that any considerable number of the many and varied cases demanding medical treatment, and occurring in the practice of any one physician, have their origin in a morbid condition of the teeth and surrounding parts, but that cases do occur, presenting diseased conditions, from the mildest to the most complicated and severe, whose origin and continuance can be traced to a morbid condition of the teeth and gums, is a truth susceptible of abundant proof; and why is it that some medical men should be so unmindful of the fact, is to me not a little surprising.

Within the last few years, not a little has been published in our dental journals, on the real and smypathetic relations which exist between the diseases of the teeth and contiguous parts and the general system, and urging upon physicians the duty of oftener inquiring into the conditions of these organs, to see whether they are not the cause of much of the functional disturbance and organic disease which they are called upon to treat. But from the very *limited* number of dental journals taken by medical practitioners, and from the very little that I have found, in my limited reading of medical journals, written upon this subject, it is to be feared that very little is ever read by the medical profession upon this point.

This is to be regretted, for I am confident that if more attention were paid to this, many physicians would better appreciate the importance of the pathological influence of these organs, saving themselves much annoyance, and their patients much suffering.

You will all acknowledge that dentition, a *physiological* process, does exert a powerful and sometimes dangerous influence over the other organs and functions of the body; and if this be so, surely their pathological or *diseased* condition cannot be less affective.

Of the evils which may result from the various morbid conditions of the teeth and gums, you will hardly expect me to do more than simply allude to some of the more important, and in the fewest words possible, to suggest the manner in which they may be produced. To attempt to define particular diseases, remote from these organs, which may depend upon their diseased condition, would be taxing your patience too much. Like the varied and multiplied causes everywhere existing, which produce disease, they usually operate upon the system in a general manner, through the medium of its nervous and vascular connection, and by the vitiated secretions which result from their own morbid condition. Every physician is aware how slight and insignificant are the causes that sometimes disturb healthy action—oftentimes so trivial as to be thought unworthy of attention—and yet such is the intimate relation which one part of the system sustains towards every other part, that the slightest departure from health in one, is almost certain to be followed by a sympathizing disturbance in every other. And when we remember the many important structures of the mouth—its nervous and vascular connections, its glands and secretions, its office in the mastication and preparation of our food for the stomach, and its intimate relation with a healthy condition of that organ—we have presented to us, in its several parts, functions the most important in their character, and a source of power over the general health, which should ever claim the watchful attention of the medical attendant.

In another part of this paper, I have spoken of the decay of the teeth, only with reference to the *causes which produced it.* Among the most common evils attendant upon decayed teeth, is what is known as *toothache,* occasioning oftentimes the most severe suffering; and after the destruction of the pulp, the contiguous parts are often involved in inflammatory action, occasioning a general disturbance of the whole nervous system, and, if long continued, more or less functional derangement; for no degree of *nervous irritability* or *excitement* can long exist, without, in some degree, being prejudicial to a healthy action of the various organs of the body.

Cases illustrative of this, I presume, are of almost daily occurrence in the experience of every medical man, in full practice, where the primary or secondary cause was to be found *only* in the unnatural influence of a morbid condition of the nervous system. The intimate sympathetic relation which exists between the uterine functions and other parts of the body, and the various and widely different manifestations of functional and constitutional suffering which certain of its physiological and pathological conditions induce, are well known; and why may not diseases of the teeth, and their appendages, if not equally prejudicial in their results, be equally *positive* in their effects?

Among the "thousand ills to which flesh is heir to," there are perhaps none whose causes are so little understood, and their cure so difficult as those of the nervous system, and among this class of affections, I may mention *convulsions,* which are sometimes produced by an irritability of the dental tissues. We can refer to well authenticated cases of this kind, where cures have followed the treatment of the dental structures alone. And I doubt not many more of the class of affections known as "*nervous*," might claim from physicians a like acknowledgment of their paternity, if their true cause could be known.

Under my observation have occurred some cases of this character, where, after months and years of suffering, and long-continued treatment, in the vain attempt to regain lost health, it was only recovered by having the attention called to the condition of the teeth, and their appendages, as acting a very important part in the disturbance; and after restoring these parts to healthy action, the general health was recovered.

Among these, as showing the importance of giving careful attention to these organs, I will mention the following:

CASE 1.—Miss F., daughter of a very respectable physician, was taken sick when she was three years of age; partially recovered; had a relapse; was taken with convulsions; partially recovered again; had another relapse; and was thus alternately better and worse until five years of age, during which time she ceased to grow, and became quite emaciated. Meanwhile, her father and other physicians exhausted their skill in their endeavors to relieve her case, but all with no apparent benefit.

I happened to allude, while in conversation with him, to the benefit which a lady patient of his had derived from my extracting a number of bad teeth for her six months previous. He remarked that Florence's teeth were badly diseased, and asked if I supposed they could have any material effect upon her health. I answered, that as I had not seen her, I could not tell. At his invitation, I called upon her, and found her not able to walk, and in a very emaciated condition. Upon examining her mouth, I found her six front upper teeth and one molar on each side decayed off to the gums, the gums badly swollen, and the mucous membrane of the roof of the mouth in an excited condition. I gave, as my opinion, that whether the condition of her teeth had been the original cause of her sickness or not, it was not impossible that they acted a very important part in keeping her in her present condition, and advised their extraction. On removing them, large quantities of pus followed, and I found the alveoli very much diseased. Upon inquiry, I found that, during her two years' illness, she had been troubled with toothache and swelled face much of the time. After the extraction of the teeth, she began to recover, and without the aid of medicine regained perfect health.

CASE 2.—Mrs. S., of this city, called on me in March, 1856, in very poor health. She had been so much of an invalid for several years, that she had been able to do but very little. She told me she had doctored a great deal, but without avail. I found her teeth and gums in a bad condition. Her teeth were past saving, and I accordingly extracted eleven. She called on me again in three months, and her health was so much improved, and she had gained so much in flesh, that I hardly knew her; and she is now, without the aid of medicine, perfectly restored to health.

I could give numerous instances, coming under my own observation, and others from reliable sources; but these will suffice to illustrate this matter.

Intimately connected with the teeth and their appendages, is the stomach and its functions. The process of digestion is commenced in the one, and nearly completed in the other, and to its perfect accomplishment, a healthy condition of the body is requisite. Nature never designed the teeth for mere *ornament*, nor the secretions of the mouth as simply fluids to moisten our food for an easy deglutition. They have other purposes to serve in the animal economy, —the one, that of mastication of the food, and the other, that of an essential constituent in the process of digestion, and it follows, as a legitimate inference, that any departure from a healthy condition of these organs, or any change in the condition of these fluids, from what is normal, must sooner or later weaken the digestive powers, and favor the establishment of dyspepsia and its attendant evils. Much more might be said in support of this argument, but your intelligence renders it unnecessary.

I wish to allude once more to a subject, to which reference has been made, and I have done. I refer to the inhalation of air, rendered *impure* and unwholesome from a want of cleanliness and care of the teeth and gums. If "cleanliness is akin to godliness," surely the door out of which our thoughts go forth, and through which air for our lungs, and the rations for the body enter, ought to be kept clean. The vile smells which greet our noses, from some mouths, are any thing but good evidences of a cleanly mind. Nor is this condition confined exclusively to the lower walks of society. Many persons who expend ample time in dressing and adorning their outward persons, and lay great claim to respectability, brush their teeth but "*semi-occasionally;*" and when this duty is performed, it is so imperfectly done, that it is but seldom that anything more than their *front* teeth are touched; and their back teeth are left in such a disgusting condition, that we have ample reason to doubt their having a good title to the claim of gentility. It is an every-day experience to meet persons, who, from infancy, have no more thought of brushing, or otherwise cleansing these organs, than if such a thing were unknown. This neglect results in an accumulation of food, tartar, and other matter, between and around the teeth, undergoing decomposition and change, which, added to the offensiveness of the morbid state of these parts, makes up such a "*combination* of villainous *smells*," that it would be difficult to imagine *any place*, the odor of which would not be a *relief*. And the medical and dental professions, as guardians of the public health, will have accomplished much when they shall have taught the people to keep their mouths clean. To talk of sending such persons to the sea shore for pure air, without properly treating their decayed teeth, and otherwise purifying their mouths, is simply *nonsense*. It would have been as easy for the hosts of Pharaoh to pass through the Red Sea *dry* as for pure air to reach the lungs through such a "*pass*" uncontaminated with pestilential vapors. And how such persons can *live* is to me a great mystery; but I *suppose* it is all in "knowing how," and it is a singular illustration of the power of the "*force of habit.*" But justice does not always sleep, and such persons will sooner or later pay the penalty of outraged decency and their defiance of the laws of health.

Thanking you for your kind indulgence and attention during the reading of what, I fear, has been an uninteresting paper, I will take my leave of the subject, amply remunerated if I have succeeded in suggesting a useful thought to your minds upon the importance of giving the "*diseases*

*of the teeth,"* in your intercourse with your patients, your careful and earnest consideration.

Gentlemen, our professions are different, and our fields of labor separate, and yet they depend upon each other; and he is the better prepared to practice the one who has an intelligent understanding of the fundamental principles which guide the practice of the other.

